# TFpredict and SABINE: Sequence-Based Prediction of Structural and Functional Characteristics of Transcription Factors

**DOI:** 10.1371/journal.pone.0082238

**Published:** 2013-12-12

**Authors:** Johannes Eichner, Florian Topf, Andreas Dräger, Clemens Wrzodek, Dierk Wanke, Andreas Zell

**Affiliations:** 1 Center of Bioinformatics Tuebingen (ZBIT), University of Tuebingen, Tübingen, Germany; 2 University of California San Diego, La Jolla, California, United States of America; 3 Center for Plant Physiology Tuebingen (ZMBP), University of Tuebingen, Tübingen, Germany; Cincinnati Childrens Hospital Medical Center, United States of America

## Abstract

One of the key mechanisms of transcriptional control are the specific connections between transcription factors (TF) and *cis*-regulatory elements in gene promoters. The elucidation of these specific protein-DNA interactions is crucial to gain insights into the complex regulatory mechanisms and networks underlying the adaptation of organisms to dynamically changing environmental conditions. As experimental techniques for determining TF binding sites are expensive and mostly performed for selected TFs only, accurate computational approaches are needed to analyze transcriptional regulation in eukaryotes on a genome-wide level. We implemented a four-step classification workflow which for a given protein sequence (1) discriminates TFs from other proteins, (2) determines the structural superclass of TFs, (3) identifies the DNA-binding domains of TFs and (4) predicts their *cis*-acting DNA motif. While existing tools were extended and adapted for performing the latter two prediction steps, the first two steps are based on a novel numeric sequence representation which allows for combining existing knowledge from a BLAST scan with robust machine learning-based classification. By evaluation on a set of experimentally confirmed TFs and non-TFs, we demonstrate that our new protein sequence representation facilitates more reliable identification and structural classification of TFs than previously proposed sequence-derived features. The algorithms underlying our proposed methodology are implemented in the two complementary tools TFpredict and SABINE. The online and stand-alone versions of TFpredict and SABINE are freely available to academics at http://www.cogsys.cs.uni-tuebingen.de/software/TFpredict/ and http://www.cogsys.cs.uni-tuebingen.de/software/SABINE/.

## Introduction

Transcription factors (TF) are the key regulators of cell- and tissue-specific regulation of gene expression and play a crucial role in the orchestration of diverse biological processes, such as cell differentiation and the adaptation to changed environmental conditions [1]–[3]. The induction or activation of target genes is achieved by the specific recognition of a DNA-motif located in the corresponding promoter regions, which is specifically recognized by the DNA-binding domain(s) of a TF. The specific interactions between TFs and their target genes are of high relevance for a more profound understanding of transcriptional gene expression in eukaryotes.

As experimental techniques for characterizing the structure and determining the binding sites of TFs are expensive and mostly performed for individual factors only, there is a need for accurate computational methods, which can be employed in large-scale studies aimed at the genome-wide analysis of transcriptional regulation in eukaryotic model organisms.

Within the last years, diverse computational approaches have been developed for the identification and structural characterization of TFs. In most cases, these methods rely on sequence homology and use heuristic alignment methods (e.g., BLAST or PSI-BLAST) to identify proteins, which are structurally similar and functionally related to a given input protein [4], [5]. Besides these alignment-based methods, diverse machine learning-based methods have been designed for protein sequence classification. All of these approaches involve the computation of a numerical feature representation of the input sequence and the training of a supervised classifier on a set of labeled sequences. For the feature representation approaches using the single amino acid composition, the dipeptide composition or a pseudo-amino acid composition have been proposed [6], [7]. More recently, PSSM profile features generated by PSI-BLAST, or functional domain composition features generated by InterProScan were successfully employed for characterizing proteins with respect to functional classes, subcellular locations, or certain structural properties [8]–[11]. Among many other supervised learning algorithms, artificial neural networks, SVMs, and HMMs were adopted to train abstract models for the prediction of protein attributes based on various features derived from their amino acid sequences [12]–[14].

The problem of predicting TF binding specificities has also been previously addressed by various studies employing diverse computational approaches. The pioneering methods in this field infer deterministic rules from the empirical inspection of protein-DNA complexes, i.e., individual amino acid binding preferences are deduced from the observed frequencies of contacts with specific nucleotides [15]. Tan *et al.* propose a comparative genomics approach to connect novel TFs with DNA-binding motifs in *E. coli* [16]. More recently, Morozov *et al.* inferred position frequency matrices (PFMs) from protein-DNA complexes and predicted binding sites of 67 TFs of *S. cerevisiae* using a Bayesian Gibbs sampling algorithm in combination with structural homology models [17].

Here, we present a four-step classification workflow for the structural and functional characterization of TFs, which combines homology-derived knowledge with supervised learning techniques for the prediction of protein characteristics. Given a set of protein sequences, our method 1) identifies the TFs among these sequences, 2) predicts their structural superclasses, 3) identifies their DNA-binding domains, and 4) infers their DNA motif. The first two steps are performed using machine learning-based classifiers trained on a novel feature representation for protein sequences, which is here demonstrated to increase the prediction accuracy of common supervised classifiers when compared to feature types proposed in preceding related studies. In the third step known protein domains are detected in the input sequences using the tool InterProScan and the DNA-binding domains are selected based on specific GO terms attributed to these domains. Finally, a recently published algorithm adopting Support Vector Regression (SVR) is used to identify TFs with similar DNA-binding specificities based on their DNA-binding domain sequence similarity to the input TF [18]. We improved the algorithm in order to increase the chance that a PFM can be transferred to a given TF. Furthermore, the prediction accuracy has been evaluated depending on diverse types of sequence similarity scores that are used as predictive features for SVR model construction.

The described algorithms are implemented in the user-friendly tools TFpredict and SABINE, which are freely available to academic researchers. For convenience, both tools were integrated into a web-based bioinformatics pipeline for the structural and functional annotation of transcription factors.

## Methods

### Generation of the validation datasets

First, we obtained protein sequences of TFs from the expert curated databases TRANSFAC (version 2012.2) and MatBase (version 8.2) [19], [20]. If a TF was contained in both databases the corresponding entries were merged in order to generate a non-redundant dataset. For each TF entry, we extracted the corresponding EntrezGene ID, organism, and PFMs. The protein sequences were obtained from UniProt, and missing superclass annotations were taken from TRANSFAC [21], [22]. Protein domains were either extracted from UniProt or predicted using InterProScan (version 4.6). Subsequently, these domains were filtered using a predefined set of GO terms (all child terms of “DNA-binding”), in order to specifically filter DNA-binding domains. Then, 41,622 non-TF proteins were extracted from UniProt (release 2012_06), using the key words *kinase, ubiquitin, actin, antigen, biotin, histone, chaperone, tubulin, transmembrane protein, endonuclease, exonuclease, translation initiation factor* in the database query, as previously done by Zheng *et al.* [14], [23]. In order to reduce the chance of mislabeling errors and thereby, ensure sufficient quality of our validation set, we refined the automatic labeling procedure proposed by Zheng *et al.* by adding diverse post-filtering steps. First, we removed all obviously mislabeled non-TFs, for which entries existed in the TF databases TRANSFAC and MatBase. Second, we excluded all putative non-TFs that were associated to the TF-specific GO term “sequence-specific DNA binding transcription factor activity” (GO:0003700). Due to these post-filtering steps, 374 (2.6%) non-TFs that are expected to have a high chance of being mislabeled could be identified.

In order to reduce the redundancy among the 3,340 TF sequences, we employed the clustering tool CD-HIT, which selected 1,487 representative sequences using a sequence similarity threshold of 80% [24]. The number of non-TFs was then chosen based on the ratio 1∶10 for TFs to non-TFs, which we would expect in eukaryotes based on previous studies [25], [26]. Using a sequence identity threshold of 56% in CD-HIT, we obtained a strongly homology-reduced set of 14,814 non-TFs. On the basis of the compiled sets of proteins, we evaluated the prediction accuracy of different classification steps, namely TF/non-TF classification, superclass prediction and PFM inference. Finally, we excluded all proteins for which no domains could be identified by InterProScan or for which no BLAST hits were reported, because some of the feature types considered in our comparison could not appropriately represent these proteins. Consequently, our final validation set contained 1,485 TFs and 14,032 non-TFs. After subdividing the TF sequences based on their structural superclasses, the validation set encompassed 271 Basic domain, 228 Zinc finger, 787 Helix-turn-helix, 101 Beta scaffold, and 98 other TF sequences. Dataset S1 contains all TF and non-TF sequences in FASTA format.

### Generation of BLAST score percentile features

In order to combine the benefits of homology-based approaches with robust machine learning-based prediction models for the identification and classification of TFs, we conceived a novel feature representation for primary protein structures, which captures their homology relations to proteins of known class. For this purpose, we developed the BLAST score percentile features, which enable the incorporation of the results from a BLAST search into state-of-the-art machine learning methods for supervised classification. The idea is to transform a BLAST result, i.e., a list of hits each associated with a bit score, into a feature vector of fixed size. The bit score 
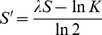
 is derived from the raw score *S* and the constants *K* and *λ*, which depend on the used scoring system which is in our application provided by the BLOSUM62 substitution matrix [27]. The raw score *S*, which equals the sum of BLOSUM62 substitution matrix scores across all amino acid pairs aligned between the query and hit sequence is converted into a normalized bit score, which is comparable between different scoring schemes [27]. In this work, the bit score distributions obtained for the hits of each protein class (TF and non-TF) were represented by means of percentiles. For TF/non-TF classification, we computed the minimum, lower quartile, median, upper quartile and maximum of the bit scores assigned to BLAST hits which are known TFs. Analogously, a 5-dimensional percentile feature vector was computed to capture the bit score distribution of the non-TF hits. The final feature vector is then obtained by concatenation of the two class-specific components. For superclass prediction the protein sequences were mapped to 25-dimensional feature vectors composed of 5 components, each corresponding to a certain superclass and capturing the specific bit score distribution observed for the respective hits.

### Generation of PSSM profile features

The use of homology-based features for the detection of DNA-binding proteins, such as TFs, was previously proposed by Kumar *et al.*, who introduced a PSSM-based feature representation, which exploits evolutionary information for classification of protein sequences [28]. First, the tool PSI-BLAST is used to generate a position-specific scoring matrix (PSSM) built from the hits of a local alignment of the query sequence against a sequence database. The iterative PSI-BLAST heuristic identifies proteins containing similar subsequences and merges these hits into an *n*×20 PSSM profile, which is refined until convergence in a predefined number of further BLAST runs. In order to transform the PSSM into a feature representation with a fixed number of dimensions, the *n*×20 PSSM is transformed into a 20×20 matrix by summing up rows that correspond to same amino acid. After dividing each value by the sequence length and scaling the result using the function 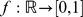 with 
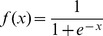
 the modified 20×20 matrix is written as a 400-dimensional feature vector.

### Generation of *k*-mer features

Another approach, which is in wide and common use for the numerical feature representation of amino acid sequences, is based on the presence or frequency of subsequences of length *k*. The basic idea of these *k*-mer features is to represent a sequence by the subsequences of length *k*, which are contained therein. For a fixed *k*, a feature vector containing 20*^k^* components is generated, where each component represents a certain *k*-mer. Then the number of occurrences of each *k*-mer is used as feature representation for a given protein sequence [29].

### Generation of pseudo amino acid features

The naive approach for obtaining a feature representation of a protein sequence is to use the relative frequencies of each amino acid. As this trivial feature type does not capture any information on the sequence order, the pseudo amino acid features were proposed as a complementary feature type [6]. In essence, these features score the physicochemical similarities Θ(*R_i_, R_j_*) of two contiguous amino acids *R_i_* and *R_j_*. These correlation scores are then averaged across all pairs of amino acids which have a distance *i* 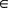 {1,…, *λ*} to each other in order to compute the *i*-th tier correlation factors *θ_1_*, …, *θ_λ_*. After a final scaling step a feature vector containing 20 features capturing amino acid frequencies and *λ* features incorporating the sequence order is generated.

### Generation of functional domain composition features

An approach, which was successfully used by Zheng *et al.* for the detection and classification of TFs, is to represent a protein sequence by its domain composition [11]. Known protein domains and functional sites within the given sequence can, for instance, be identified based on profile-HMMs using the tool InterProScan [30]. A sequence is then represented by a binary feature vector containing a component for each protein domain that was found in one of the training sequences. If a certain domain was found in the input sequence, the respective component is set to 1 and otherwise to 0.

### Classification of protein sequences

Different protein sequence representations were incorporated as features into the classifiers SVM, KNN, Naive Bayes, Decision Tree and Random Forest implemented in the WEKA package [31]. A 4×4-fold nested cross-validation was performed in order to assess the classification performance. To this end, the validation data was split by stratified sampling, in order to maintain the original class distribution in each subset. Model parameters were tuned in an unbiased manner by performing an inner cross-validation on the training data of each outer cross-validation split. A summary value of the classification performance was then obtained by calculating the average area under the ROC curve (avgROC). As the avgROC is only applicable to assessing the performance of binary classifiers, we split the superclass prediction task into five one-versus-rest classification tasks and averaged the corresponding ROC scores.

### Superclass prediction method by Zheng *et al.*

In contrast to our approach, which combines five binary one-versus-rest classifiers for the discrimination between five structural superclasses of TFs, the state-of-the-art method proposed by Zheng *et al.* employs the concept of Error-Correcting Output Codes (ECOC) for constructing a multiclass meta-classifier on top of multiple two-class SVMs. According to Dietterich *et al.*, who first described this methodology, a code of length 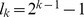 is required to solve a *k*-class problem [32]. The code length 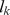 directly corresponds to the number of binary classifiers needed to solve the multiclass learning problem. Hence, Zheng *et al.* had to integrate the prediction outcomes of seven SVMs for distinguishing between the four main superclasses: Basic domain, Zinc finger, Helix-turn-helix and Beta scaffold [14], [21]. Since in this work we also account for the fifth superclass “Other”, we extended the Zheng method to the five-class learning problem evaluated here, which requires the training of 15 binary SVM classifiers. Following the definition of ECOC given by Dietterich *et al.*, we constructed an exhaustive code for five classes as illustrated in Table 1 [32]. Positively and negatively labeled TFs were determined for each SVM according to the bit strings corresponding to the columns of Table 1. In order to predict the class of an input protein sequence, the binary outcomes of the 15 classifiers were concatenated to a bit string of length 15, and the hamming distance to each row in Table 1 was calculated. As each row corresponds to a certain superclass, the predicted class can be obtained from the row with the shortest hamming distance. If the row could be unambiguously determined by minimum distance decoding, a probability of 1 was assigned to the corresponding class and all other classes were given a probability of 0. If multiple rows with minimum distance existed, we assigned equal probabilities to each of the corresponding classes.

**Table 1:** Exhaustive Error-Correcting Output Code for TF superclass prediction.

	SVM classifier														
Superclass	1	2	3	4	5	6	7	8	9	10	11	12	13	14	15
Other	1	1	1	1	1	1	1	1	1	1	1	1	1	1	1
Basic domain	0	0	0	0	0	0	0	0	1	1	1	1	1	1	1
Zinc finger	0	0	0	0	1	1	1	1	0	0	0	0	1	1	1
Helix-turn-helix	0	0	1	1	0	0	1	1	0	0	1	1	0	0	1
Beta scaffold	0	1	0	1	0	1	0	1	0	1	0	1	0	1	0

The table shows the code used for the construction of a 5-class ECOC classifier which integrates the prediction outcomes of 15 binary SVM classifiers. Each column corresponds to a two-class SVM, which treats structural classes assigned to 1 as positives and classes assigned to 0 as negatives. The rows correspond to the 5 superclasses. Each entry (bit) in the table equals to the binary prediction outcome expected from a certain SVM classifier for a query protein of a specific superclass.

### Identification of DNA-binding domains

The stand-alone version of the tool InterProScan (http://www.ebi.ac.uk/Tools/pfa/iprscan) was downloaded from EMBL-EBI and locally installed. All protein signature recognition methods provided by InterProScan were used to predict functional domains. Default parameters were used, except that the option for returning Gene Ontology (GO) terms for each identified protein domain was switched on. Based on the GO term associations the DNA-binding domains among the domains returned by InterproScan could be filtered. For this purpose, a list containing the GO identifiers of the molecular function “DNA-binding” and of all sub-categories was obtained from the GO project website (http://www.geneontology.org).

### Inference of DNA motifs

**Figure 1:**
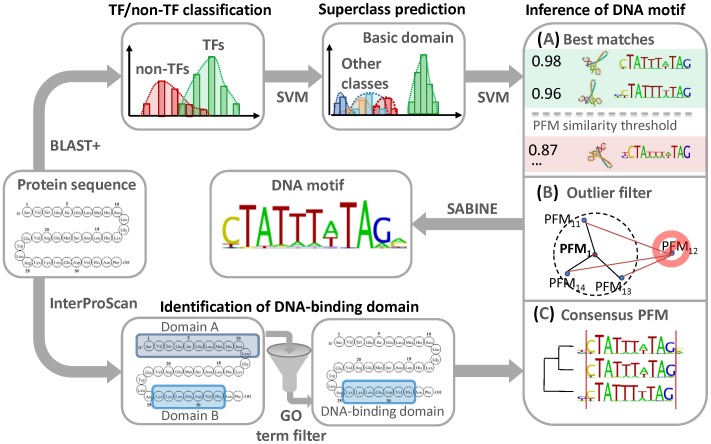
Bioinformatics pipeline for the structural and functional annotation of transcription factors. First the input protein sequence is aligned to a non-redundant protein database using the BLAST heuristic. The bit score distributions of the TFs and non-TFs among the BLAST hits are represented by means of percentiles. These percentiles are incorporated into SVM classifiers for the discrimination of TFs from non-TFs (Step 1). If a given protein sequence was classified as a TF, another SVM is applied to predict its structural superclass (Step 2). The tool InterProScan is used to predict the functional domains of the TF and the DNA-binding domains among these are identified based on the associated GO terms (Step 3). Finally, the tool SABINE infers a DNA motif using an SVR-based algorithm (see Methods section) that takes the structural superclass and DNA-binding domains of the TF as input (Step 4).

The prediction of the DNA motif that is specifically recognized by a given TF was performed based on the improved version of a recently published algorithm, which is implemented in the current version of the tool SABINE [18]. SABINE (Stand-Alone BINding specificity Estimator) compares a given transcription factor to a predefined set of TFs for which experimentally confirmed DNA motifs are available from appropriate databases (e.g., TRANSFAC, MatBase or JASPAR) [19], [20], [33]. Based on various features capturing evolutionary, structural, and physicochemical similarities of the DNA-binding domains, the PFM similarity is predicted by means of Support Vector Regression (SVR). The best matches, i.e., the TFs with highest predicted PFM similarity to the factor of interest, are filtered based on a predefined PFM similarity threshold (Figure 1A), which is dynamically chosen in the current version of SABINE. Then an outlier filter is applied, which avoids the merging of dissimilar PFMs. By filtering PFMs with high relative average distance to the other matrices, the filter ensures homogeneity of the remaining PFMs (Figure 1B). The merging of the PFMs is performed using a progressive alignment algorithm implemented in the tool STAMP, which successively aligns the DNA motifs of the best matches along a guide tree (Figure 1C). The resulting consensus binding profile of the filtered best matches corresponds to the predicted DNA motif.

### Assessment of the PFM transfer error

The correspondence between the predicted and experimentally confirmed PFMs was assessed based on the *S^max^*-log-odds score, which was proposed as a PFM similarity measure by Pape *et al.* [34]. Given two PFMs *X* and *Y* the authors compute the probability *γ_X,Y_*(*k*) of a hit of *X* which overlaps at the *k*-th position with a hit of *Y* in a random DNA sequence. Next, the log ratio 
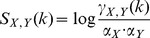
 of *γ_X,Y_*(*k*) and the joint probability 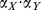 that a hit was independently created by *X* and *Y* at this position is calculated. By computing the maximum across all offsets *k* between the hits of *X* and *Y* and across all possible orders (i.e., *XY* and *YX*) and orientations (i.e., forward and reverse strand) of the DNA motifs, the log-odds score 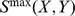 is computed according to the following equation, where 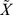 and 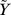 denote the reverse complements of the PFMs *X* and *Y*, respectively: 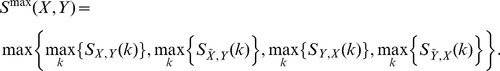



For the sake of better interpretability the PFM similarity score 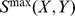 was normalized using the geometric mean of the similarities computed for the comparison of *X* and *Y*, respectively, against itself: 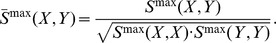



Finally, the normalized PFM similarity score was converted into a distance 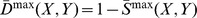 defined on the interval [0, 1] which was used to assess the PFM transfer error, i.e., the deviation between the predicted and experimentally confirmed DNA motif of a TF, in a 4-fold stratified cross-validation procedure.

### Implementation of TFpredict

**Figure 2:**
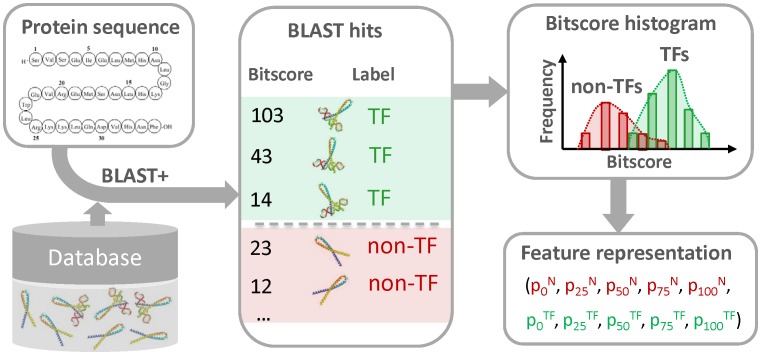
Calculation of BLAST bit score percentile features. The protein sequence is aligned to TF and non-TF sequences in a non-redundant sequence database, which does not contain the input sequence itself. Next, the bit scores of all TFs and non-TFs among the BLAST hits are extracted from the BLAST result. The bit score distributions observed for TFs and non-TFs, respectively, are represented based on the minimum *p_0_*, the lower quartile *p_25_*, the median *p_50_*, the upper quartile *p_75_* and the maximum *p_100_*. The bit score feature representation is then obtained by concatenation of the components calculated for the TF and non-TF class. In addition to binary classification tasks this feature representation is also applicable to multiclass problems, such as the prediction of TF superclasses. For this purpose, the feature vector components capturing the bit score distributions of each superclass were concatenated.

As SABINE requires the structural superclass, and the DNA-binding domains of a given input TF, we developed the tool TFpredict, which infers all structural properties of TFs needed by SABINE. As described in more detail above, these characteristics are obtained from SVM classifiers trained on BLAST-based features (Figure 2) and from the tool InterProScan. TFpredict is completely implemented in Java™. It uses the online version of InterProScan for DNA-binding domain identification and employs classifiers from the Weka package for TF/non-TF classification and superclass prediction [30], [31]. In order to more effectively exploit the domain information acquired from InterProScan, the user can choose if predictions shall be made based on characteristic domains, which were specifically observed in TFs of a certain superclass during training. The calculation of the bit score percentile features, used for numerical protein sequence representation, involves the use of the sequence similarity search tool BLAST+ [35]. TFpredict runs out of the box on any system, provided that a Java**™** virtual machine is installed.

### Implementation of SABINE

SABINE is also implemented in Java, but requires a Linux platform, as it depends on diverse platform-dependent third-party software packages. For domain sequence similarity estimation, SABINE uses alignment methods implemented in BioJava as well as several sequence kernels (e.g., Local Alignment Kernel, Mismatch Kernel, SVM-pairwise score) [29], [36]–[39]. To estimate the structural domain similarity of TFs, we integrated PSIPRED to predict secondary structures [40]. We measure PFM similarity using MoSta and for the merging of best match PFMs the tool STAMP is applied [34], [41]. For training and evaluation of the Support Vector Regression models the libSVM implementation was used [42]. Since the last release, SABINE was enhanced with various features, which simplify the usage and increase the applicability of the tool. A graphical interface and a publicly available online version of SABINE were implemented. Furthermore, a convenient installation script is provided for the stand-alone version of the tool. The best match threshold parameter (see Methods section) is now dynamically chosen, depending on the quality of the best matches, such that there is an increased chance that a DNA motif can be predicted for the given input TF. Furthermore, a confidence is now associated with each prediction.

## Results and Discussion

### Discrimination of TFs from other proteins

**Figure 3:**
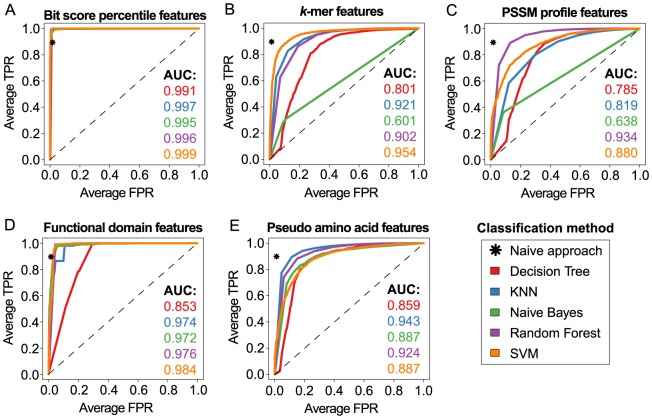
Evaluation of classifiers and feature types for TF/non-TF discrimination. (**A**) Each of the shown curves corresponds to one of five supervised machine learning methods trained on our novel bit score percentile features, which were employed to distinguish TFs from other proteins. The individual curves obtained for each of the four cross-validation folds were averaged based on the class discrimination cutoffs. Averaged ROC curves were computed in an analogous manner for (**B**) *k*-mer features, (**C**) PSSM profile features, (**D**) functional domain features and (**E**) pseudo amino acid features. The sensitivity and specificity achieved by the naive BLAST-based approach correspond to a single point in ROC space marked by an asterisk.

We evaluated the performance of different sequence-based feature types for the prediction of relevant structural and functional attributes of specific transcription factors. Seizing and combining the main concepts of homology-based and machine learning-based approaches, we created a numerical sequence representation, which enables the accurate discrimination of TFs from other proteins (Figure 2). In a comparison against published protein sequence representations in terms of the average performance achieved by widely used supervised classification methods, our bit score percentile features were ranked first (Figure 3). The performance was assessed on a homology-reduced set of protein sequences, containing 1,485 TFs and 14,406 non-TFs, based on the average area under the ROC curve resulting from a 4×4-fold nested, stratified cross-validation.

From Figure 3 it becomes apparent that most feature types achieve less sensitivity (<0.9) at an equally high specificity of 0.98 when compared to the naive method that simply predicts the class of the best BLAST hit among the homology-reduced set of training sequences with less than 100% sequence identity to the query protein. Interestingly, only the feature types that incorporate prior knowledge about either protein function of homologues or functional domain composition are capable of achieving comparable or higher classification accuracy than the naive method. While the performance observed for the domain-based features still leaves some room for improvement and shows a stronger dependency on the chosen machine learning technique, a nearly perfect classification outcome was found for the bit score percentile features independent of the employed classifier. It is also notable that the TF prediction performance of pseudo-amino acid features is increased for most classifiers when compared to PSSM profile and *k*-mer features, respectively. This result may in part be explained by the fact that pseudo-amino acid features account for the sequence order to some degree while this information is completely lost by PSSM compression and *k*-mer fragmentation, respectively. The average performance measured in terms of ROC score for each classification method is depicted in Figure S1. In summary, SVM, KNN and Random Forest, which achieved a mean ROC score (avgROC) greater than 0.93, performed considerably better on the task of separating TFs from non-TFs than Naive Bayes (avgROC  = 0.82) and Decision Tree (avgROC  = 0.86).

For all approaches that involve the use of BLAST (i.e., the naive method, the PSSM profile and the bit score percentile features) a prediction is only possible, if at least one hit with sufficient sequence similarity to the input protein is detected in the sequence database. This issue prevented us from making a prediction for 14 (0.1%) of the proteins in our dataset and was only observed for non-TFs, as a consequence of the more stringent similarity threshold (80% for TFs and 56% for non-TFs), which was used to reduce sequence redundancy. However, a considerably higher limitation of the prediction rate was observed for the functional domain features. As InterProScan was not able to identify any known domains for 397 proteins, a prediction was not possible for 2.4% of the sequences. In summary, the comparison of the two feature representations, which permitted higher classification performance than the naive approach, showed that our novel bit score percentile features offer a significantly higher chance that a prediction is possible than the functional domain features.

### Prediction of transcription factor superclasses

**Figure 4:**
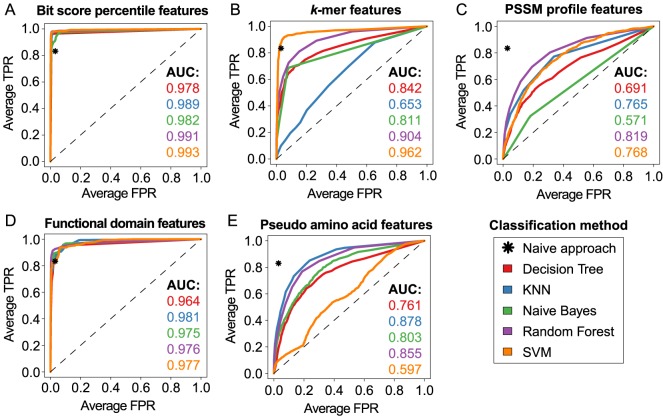
Evaluation of classifiers and feature types for superclass prediction. The classification performance of representative and widely used machine learning methods incorporating different features for superclass prediction was assessed my means of threshold-averaged ROC curves obtained from stratified 4×4-fold nested cross-validation. The differently colored curves correspond to distinct classification methods (see legend). For each classifier the area under the curve (AUC) is denoted. ROC curves were obtained from classifiers incorporating (**A**) our novel bit score percentile features, (**B**) *k*-mer features (**C**) PSSM profile features (**D**) functional domain features and (**E**) pseudo amino acid features.

In order to assess the performance achieved by different combinations of classifiers and features for superclass prediction, the ROC scores of binary one-versus-rest classifiers were determined by a 4×4 nested cross-validation for each of the five superclasses and then the average was calculated. Using the naive approach a mean sensitivity of 0.84 was obtained at a specificity of 0.97. A significantly weaker performance was observed for PSSM profile, pseudo-amino acid and *k*-mer features (Figure 4). While the achieved classification performance was independent of the classification method for the former two feature types, the accuracy achieved with *k*-mer features was particularly high when used in combination with SVM. The benefits of *k*-mers as feature representation for SVMs have been previously demonstrated by Leslie *et al.*, who first introduced the *k*-mer-based spectrum and mismatch kernels [29], [38].

As stated for TF/non-TF classification, the bit score percentile and functional domain features stand out as the most powerful numerical representations of protein sequences. Both feature types facilitate the nearly error-free assignment (avgROC >0.98) of TFs to their structural superclasses. Strikingly, less misclassification was found for sequences represented by bit score percentiles. The best average classification performance was achieved by the Random Forest and SVM (avgROC >0.9). The remaining three classifiers achieved mean ROC scores between 0.83 and 0.89 (Figure S2 B). When assessing the classification error separately for each one-versus-rest classifier trained for the specific recognition of a certain superclass, we observed that the most reliable predictions were made for the zinc finger class (Figure S2 C). Nevertheless, reliable predictions may not be possible for specific families of TFs, such as C2H2-type zinc fingers proteins. This problem arises from the fact that C2H2-type DNA-binding domains (IPR013087) do not specifically occur in Zinc finger TFs, but also in non-TF proteins with DNA-binding activity (e.g., Q9STM3, Q6P2A1) or TFs attributed to other superclasses (e.g., P15336, Q3T921), due to a structurally different, second DNA-binding domain. Consequently, approaches that infer structural TF classes based on sequence homology may fail or produce ambiguous prediction outcomes, because of the structural heterogeneity of the local alignment hits.

In order to systematically screen for problematic TF instances and families, TFpredict was applied to a large set of protein sequences, comprising 6,314 TFs with superclass annotations from TRANSFAC. The results from this global analysis can be found in Table S1. In short, 192 of 6,314 (3%) proteins were not identified as TFs and for 500 of 6,122 (8%) proteins recognized as TFs, a wrong superclass was predicted. Among the 192 unrecognized TFs 144 (75%) proteins are C2H2-type zinc fingers (2.3), which can hardly be discriminated from C2H2-type DNA-binding non-TFs, due to a high local sequence homology and similar domain composition. For all other problematic TF classes less than ten undetected TFs were observed. These classes include bZIP (1.1), bHLH (1.2), Tryptophan clusters (3.5), Homeo domain (3.1), Heteromeric CCAAT factors (4.8), Fork head/winged helix (3.3), and Runt (4.11) factors. Consistently, 308 (62%), of the 500 misclassified TFs belong to the C2H2-type zinc finger factors. We found that the high degree of misclassification observed for the C2H2-type TFs is in most cases a consequence of the structural heterogeneity of the detected BLAST hits. This heterogeneity arises from the fact that in our homology-reduced BLAST database many TFs harboring Cys2His2-type zinc finger domains are not labeled as Zink fingers, due to a second DNA-binding domain (e.g., bZIP or homeo domain), which determines their superclass. Besides many problematic factors belonging to the Cys2His2-type zinc fingers (2.3), we found that TFs from the classes HMGI(Y) (0.2.), HMG (4.7), bZIP (1.1) and bHLH (1.2) were also overrepresented among the misclassified TFs.

The sequence-based identification of TFs and their subsequent structural classification has already been performed by Zheng *et al.*, who adopted SVMs in conjunction with functional domain features for this task. Zheng *et al.* evaluated their approach by jack-knife cross-validation on a set of 138 TFs with annotated superclass, which amounts to less than one tenth of the training and validation data used in this study. In contrast to Zheng *et al.* we used nested cross-validation in order to ensure unbiased selection of optimized model parameters, and assessed the performance based on the average area under the ROC curve instead of the accuracy. Since the accuracy may be inconclusive for imbalanced datasets, we chose the avgROC, which is firstly insensitive to changes in the class distribution and secondly considers multiple thresholds for class discrimination.

### Comparison to existing methods for the identification and classification of TFs

Within the last years different tools have been developed for the sequence-based computational prediction of specific protein classes. For instance, Kumar *et al.* developed the tool DNAbinder () for the specific recognition of DNA-binding proteins. In brief, their method employs SVM classifiers trained on PSSM profile features capturing evolutionary information [28]. More recently, Zheng *et al.* implemented TFMiner that adopts an SVM classifier trained on functional domain features for TF identification and discriminates between four structural superclasses by means of multiple SVMs combined with ECOC [14].

For the comparison presented here, the methods presented earlier by Kumar *et al.* and Zheng *et al.*, respectively, were re-implemented according to the authors' descriptions, to ensure a fair performance comparison on a common validation dataset using the same randomly determined 4×4 cross-validation splits for each method. Since the web-tool DNAbinder by Kumar *et al.* was designed for the detection of DNA-binding proteins, which also includes specific families of non-TF proteins (e.g., RNA polymerases, DNA methyltransferases, etc.), a retraining of the corresponding SVM models was required for the sake of fairness. Due to the fact that Kumar's method can be straightforwardly extended to the prediction of superclasses by use of multiclass SVMs, we also investigated the suitability of this method for solving the second classification step. As Zheng *et al.* confined their analysis to four of the five possible TF superclasses, their method was also adapted to overcome its limited applicability to the here considered 5-class learning problem (see Methods).

**Figure 5:**
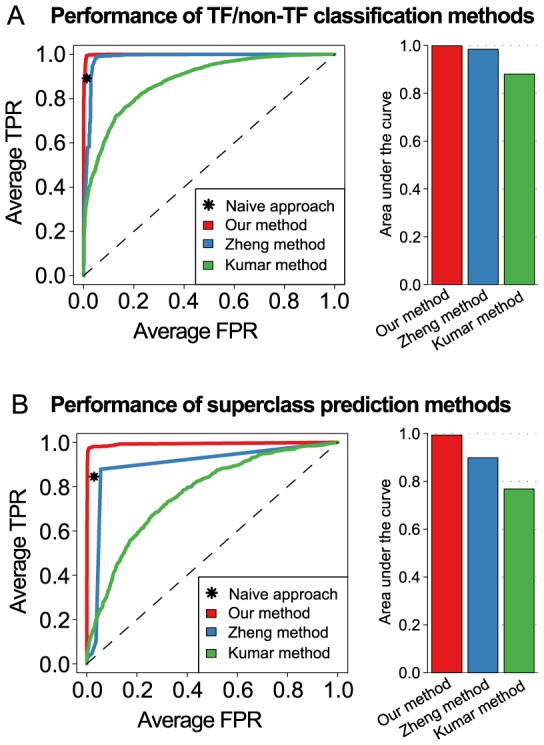
Performance comparison against previous approaches. (**A**) The classification performance achieved by our novel sequence feature representation in conjunction with SVM classifiers was compared to two other SVM-based approaches, which were previously published by Zheng *et al.* and Kumar *et al.*, respectively. The Kumar method employs SVMs trained on PSSM profile features (orange curve in Figure 3C) and the Zheng method corresponds to SVMs incorporating functional domain features (orange curve in Figure 3D). The prediction accuracy was assessed in terms of the area under the threshold-averaged ROC curves obtained from stratified 4×4-fold nested cross-validation. The bar plot beside the ROC curves depicts the area under the curve that was observed for each of the three approaches. (**B**) Similar plots as in (A), showing the results of ROC evaluation for the task of predicting the structural superclasses of TFs. Kumar's method, which was originally devised for the prediction of DNA-binding proteins, was extended to facilitate the discrimination of multiple superclasses. The corresponding ROC curve is identical to the orange curve in Figure 4C. The method by Zheng *et al.*, which was originally designed for the specific detection of 4 superclasses, was extended to the five-class problem evaluated here. As described in more detail in the methods section the extended Zheng method is based on a metaclassifier that integrates the prediction outcomes of 15 binary SVMs based on an Error-Correcting Output Codes (ECOC).

From Figure 5 it becomes apparent that only our method increases in terms of sensitivity at equal specificity as compared to the naive method. Furthermore, it can be concluded that the Zheng method clearly offers an increased performance in relation to the Kumar method. Surprisingly, the complex ECOC-based multiclass SVM (avgROC  = 0.898), which requires 3 times the computational cost, was clearly outperformed by an ordinary one-versus-rest multiclass SVM (avgROC  = 0.977, see orange curve in Figure 4D).

### Identification of DNA-binding domains

As our algorithm for DNA motif prediction requires the sequence intervals spanned by the DNA-binding domains of a given TF, the tool InterProScan which scans a given amino acid sequence using models of known domains (e.g., profile HMMs) was integrated into our four-step prediction framework. Since the tool returns the corresponding Gene Ontology (GO) terms associated to each of the identified domains, we were able to specifically filter the DNA-binding domains. We did not assess the quality of the retrieved DNA-binding domain annotations, because this information is either obtained from the external database InterPro or predicted by the tool InterProScan [30], [43]. Due to the fact that both the database and the associated scanning tool were implemented by EMBL-EBI and not modified within this work, the validation of the retrieved domain information is beyond the scope of this article and the reader is referred to the corresponding publications [30], [43].

### Prediction of transcription factor DNA motifs

In 2010, we presented a method for inferring the DNA-binding specificities of TFs [18]. On the basis of a large, non-redundant set of TFs with known binding specificities, we trained SVR-based models which quantitatively estimate the PFM similarity of two given TFs, by using diverse sequence-derived features, which measure the evolutionary, physicochemical and structural similarity of their DNA-binding domains. Thus, given a TF with unknown DNA-binding specificity, functionally similar TFs with annotated DNA motifs can be identified by using a cut-off for their predicted PFM similarity. In a second step, these PFMs are filtered for outliers, progressively aligned and then merged to generate the predicted binding profile.

Our method has been implemented in the tool SABINE, which was now equipped with a graphical user interface and trained on a large up-to-date set of TFs compiled by integration of the proprietary databases TRANSFAC (Biobase) and MatBase (Genomatix) [19], [20]. Due to diverse improvements of the algorithm, there is now an increased probability that a DNA motif can be predicted for a given TF and each prediction is associated with a confidence.

In order to increase the efficiency to the algorithm, we assessed the impact of omitting the most computationally intensive features required for predicting a PFM. From the sequence similarity features calculated by SABINE, the local alignment kernel (LAK) features and the mismatch kernel (MMK) features account for 53% and 28% of the average runtime, respectively. While a runtime of 42 min was needed on average for a prediction based on all features, 5 min were required without using LAK and MMK features on a Linux server (Scientific Linux 6.2) with AMD Opteron 6174 CPU (12 cores, 2.2 GHz). Since low runtimes may be desirable for large batch processing tasks, we investigated the performance of the SABINE algorithm depending on the inclusion of LAK and MMK features. The quality of the predicted PFMs was evaluated based on its similarity to the experimentally confirmed PFMs obtained from the source databases. To this end, we determined the PFM transfer error, i.e., the distance between the predicted and the annotated PFM, based on the *S^max^*-log-odds score (see Methods section) proposed by Pape *et al.* [34]. In parallel, we also assessed the impact of the best match threshold (BMT) parameter on the prediction accuracy and the chance that a prediction is possible. The BMT corresponds to a lower bound for the predicted PFM similarity of a best match, i.e., TF with known PFM contained in the SABINE training set, to the input factor with unknown PFM. As this parameter directly defines the set of best-matching TFs whose PFMs are merged to generate the predicted DNA motif its choice may substantially impact the quality of the results.

**Figure 6:**
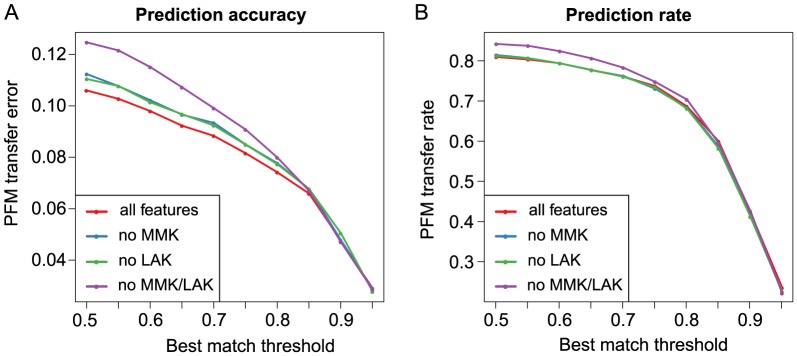
Evaluation of different features for DNA motif prediction. (**A**) The deviation between the predicted and annotated DNA motifs (i.e., PFM transfer error) was assessed based on the average [0, 1]-distance 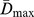 (see Methods section) by 4-fold stratified cross-validation. The curves indicate the average PFM transfer error observed for different features depending on the minimum PFM similarity (i.e., best match threshold) predicted for the training set TFs, whose PFMs were merged to generate the predicted PFM. (**B**) The relative frequency with which a DNA motif could be predicted for a given TF (i.e., PFM transfer rate) was concurrently determined for varying best match thresholds. The shown curves correspond to the PFM transfer rate observed for different features, which were incorporated into the SVR models used for PFM similarity estimation.

Consistent with our expectations it becomes clearly apparent from Figure 6 that both the PFM transfer error and the transfer rate decrease with increasing BMT. The differences in the prediction accuracies achieved with different features are most striking for lower values of the BMT between 0.5 and 0.8. When a BMT≥0.85 is selected, only marginal performance differences can be observed between features. While a high value for the BMT limits the algorithm mostly to trivial PFM transfers between homologous or closely related TFs, lower values for the BMT permit non-trivial PFM transfers, i.e., the inference of the DNA motif of a TF, for which no evolutionary related TFs with known PFM exist in the training set. With regard to the evaluation results shown in Figure 6A, one may conclude that the computationally intensive kernel-based sequence similarity measures MMK and LAK both to a comparable degree contribute to a reduced error of non-trivial PFM transfers. Consistent with these findings, we also observed a trend towards a smaller absolute average error (AAE) and mean squared error (MSE) for the superclass-specific SVR regression models, which were integrated into SABINE to estimate the PFM similarity of two TFs based on kernel- and alignment-based domain sequence similarity features (Figure S3).

In the previous version of SABINE, we proposed to use a stringent, fixed BMT of 0.95 to ensure a sufficient quality of the predicted PFMs. However, by setting the BMT dynamically at runtime based on the PFM similarities predicted for the best matches, the chance that particularly non-trivial PFM transfers are possible can be dramatically increased at the cost of a small decrease in accuracy (Figure 6B). While the old parameter settings (fixed BMT of 0.95) allowed for predicting a DNA motif for 25% of the TFs in our validation set, a PFM could be proposed for more than 80% using the new settings (variable BMT between 0.5 and 0.95). Interestingly, the introduced additional error is smaller than 0.1 in terms of normalized 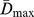 distance, which was also on average observed for PFMs determined based on different wet lab experiments [18], [34]. However, the prediction rate as well as the accuracy is expected to be lower for the public version of SABINE, as only 26% of the training data could be used.

### Availability of tools

TFpredict and SABINE are both freely available as online and stand-alone tools under GNU General Public License (GPL) version 3 and can be downloaded from http://www.cogsys.cs.uni-tuebingen.de/software/TFpredict/ and http://www.cogsys.cs.uni-tuebingen.de/software/SABINE/, respectively. For convenience, a web-based, workflow was implemented, which facilitates the sequential processing of a protein sequence with the two tools (Figure 1). The tools are fully documented and provide a command-line interface for large-scale batch processing tasks, as well as an online version, rendering the tools useful for experimental biologists.

## Conclusions

In this study we compared different features and classifiers on the problems of predicting TFs, their structural class and their *cis*-acting DNA motif, based on their amino acid sequences. For this purpose, we developed a novel feature representation that facilitates combining prior knowledge from homology searches with robust machine learning-based classification. Performing cross-validation on a homology-reduced set of labeled protein sequences, we demonstrated that our method is superior to a naive BLAST-based approach and outperforms several previously proposed sequence-derived feature representations with regard to the average prediction accuracy achieved by commonly used supervised classification methods. Our novel approach is implemented in the Java application TFpredict, which was designed as an upstream tool for SABINE, and provides all structural characteristics required by the SABINE algorithm for the prediction of TF binding specificities. Furthermore, we developed an algorithmically improved version of SABINE, which has now a considerably increased chance to succeed in making a prediction. The tool was equipped with both a GUI and web-interface and the incorporated regression models were trained on a richer and more recent set of TFs with annotated DNA motifs. A comparison of selected features of SABINE with respect to their contribution to an increased quality of the predictions showed that the inclusion of all features is in particular beneficial for non-trivial DNA motif predictions. However, if for a given factor closely related TFs with annotated DNA motifs exist in the SABINE training set, the computationally intensive kernel-based sequence similarity features MMK and LAK, which account for approximately 80% of the total run time, are not required.

In summary, the tools TFpredict and SABINE provide complementary and accurate methods for the identification, structural annotation and DNA motif prediction of TFs. Both tools can be conveniently accessed via a public web-interface and a workflow was implemented which permits the fully automated, successive execution of the tools. In summary, the algorithms and the software presented in this work contribute to a more profound understanding of the complex mechanisms underlying transcriptional regulation in eukaryotes.

## Supporting Information

Figure S1 **TF/non-TF classification performance depending on features and classifiers.** ROC scores resulting from cross-validation of different classifiers for the discrimination of TFs from other proteins are illustrated as box plots. The boxes correspond to the ROC score distributions observed for (**A**) different feature types and (**B**) diverse classification methods.
(PDF)

Figure S2 **Superclass prediction performance depending on features and classifiers.** ROC score distributions resulting from cross-validation of classifiers for superclass prediction are illustrated as box plots and were separated by (**A**) feature types, (**B**) classification methods. The boxes depicted in (**C**) correspond to the ROC scores achieved by the one-versus-rest classifiers trained for the specific detection of TFs belonging to a certain structural superclass.
(PDF)

Figure S3 **Regression error of SABINE SVR models.** Shown is the (**A**) mean squared error (MSE) and the (**B**) average absolute error (AAE) of the support vector regression models used by SABINE to predict the PFM similarity of two TFs based on diverse features measuring the sequence similarity of their DNA-binding domains. SVR models were trained separately for each of the five structural superclasses, based on TF pairs with a DNA-binding domain sequence similarity >0.3 with respect to the BLOSUM62 substitution matrix. Then, the regression error, i.e., the difference between the true and the predicted PFM similarity of each TF pair was assessed by cross-validation.
(PDF)

Table S1 **Global screen for problematic TF instances.** On the basis of the TF databases TRANSFAC and MatBase, a list of 6,314 TFs with annotated superclass was compiled. For each protein, the corresponding accession used by the source database, the gene symbol and a cross-reference to UniProt are provided. The annotated and predicted superclasses are shown in different columns for each TF. If TFpredict failed in recognizing a certain protein as a TF, the corresponding entry in the column “Predicted Superclass” was set to “NA”. The rightmost column indicates for each protein, whether the identification and structural characterization was successful.
(XLSX)

Dataset S1 **FASTAs file containing TF and non-TF sequences.** The file contains the 1,485 TF sequences and 14,406 non-TF sequences, which were extracted from the databases TRANSFAC, MatBase, and UniProt and used for classifier training and validation. For each sequence the corresponding UniProt ID, protein class (TF or non-TF) and source database (TRANSFAC, MatBase, or UniProt) is indicated in the header.
(TXT)
