# Supplementary material for: TFpredict and SABINE: Sequence-Based Prediction of Structural and Functional Characteristics of Transcription Factors
Source: PLoS One. 2013 Dec 12;8(12):e82238. doi: 10.1371/journal.pone.0082238 (PMC3861411; doi:10.1371/journal.pone.0082238)

Figure S1: TF/non-TF classification performance depending on features and classifiers

ROC scores resulting from cross-validation of different classifiers for the discrimination of TFs from other proteins are illustrated as box plots. The boxes correspond to the ROC score distributions observed for **(A)** different feature types and **(B)** diverse classification methods.

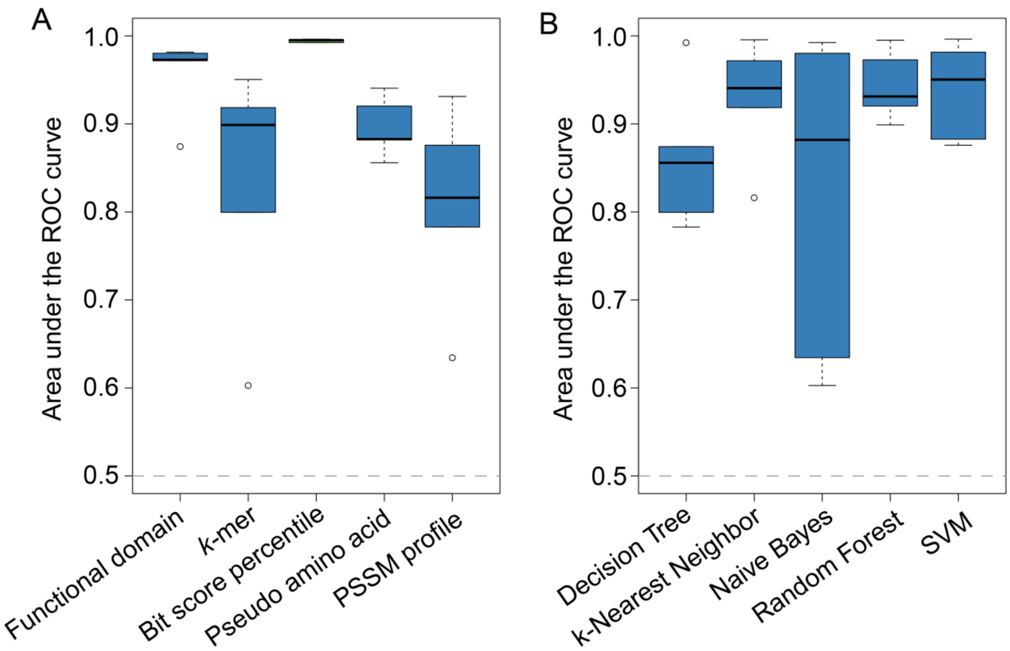

Supplement: Figure S1 — TF/non-TF classification performance depending on features and classifiers. ROC scores resulting from cross-validation of different classifiers for the discrimination of TFs from other proteins are illustrated as box plots. The boxes correspond to the ROC score distributions observed for (A) different feature types and (B) diverse classification methods. (PDF) [file pone.0082238.s001.pdf]
