# Supplementary material for: TFpredict and SABINE: Sequence-Based Prediction of Structural and Functional Characteristics of Transcription Factors
Source: PLoS One. 2013 Dec 12;8(12):e82238. doi: 10.1371/journal.pone.0082238 (PMC3861411; doi:10.1371/journal.pone.0082238)

Figure S2: Superclass prediction performance depending on features and classifiers

ROC score distributions resulting from cross-validation of classifiers for superclass prediction are illustrated as box plots and were separated by **(A)** feature types, **(B)** classification methods. The boxes depicted in **(C)** correspond to the ROC scores achieved by the one-versus-rest classifiers trained for the specific detection of TFs belonging to a certain structural superclass.

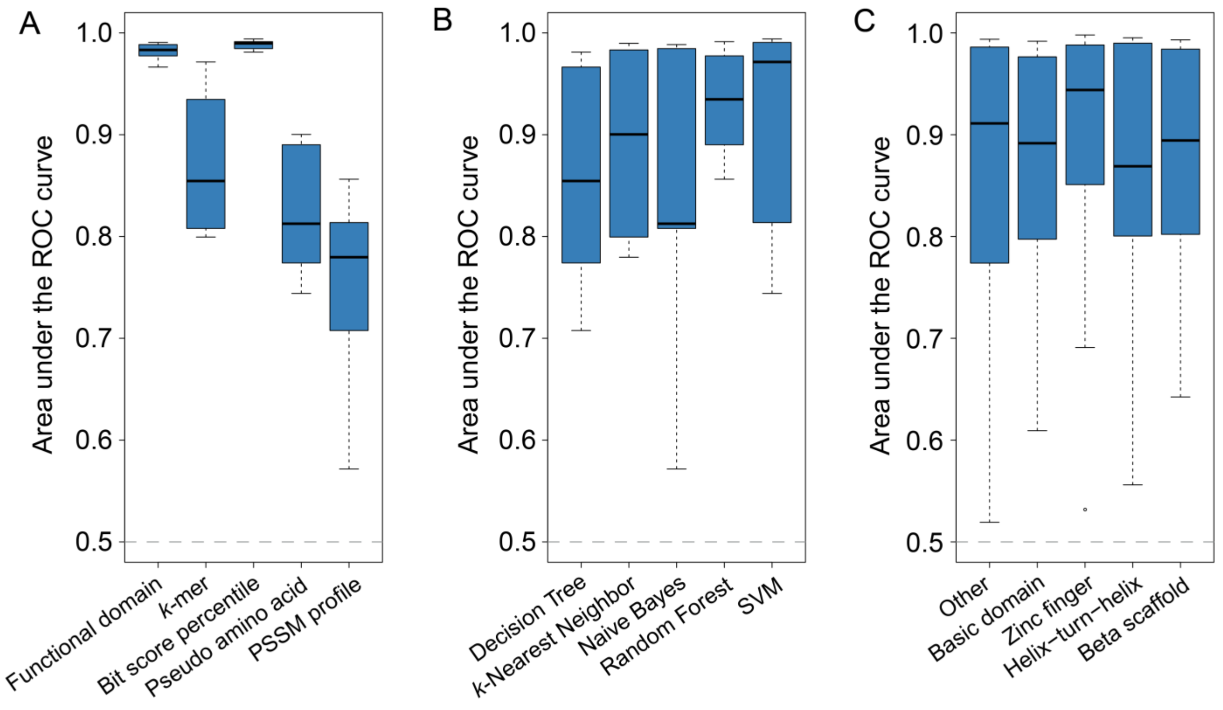

Supplement: Figure S2 — Superclass prediction performance depending on features and classifiers. ROC score distributions resulting from cross-validation of classifiers for superclass prediction are illustrated as box plots and were separated by (A) feature types, (B) classification methods. The boxes depicted in (C) correspond to the ROC scores achieved by the one-versus-rest classifiers trained for the specific detection of TFs belonging to a certain structural superclass. (PDF) [file pone.0082238.s002.pdf]
