# Supplementary material for: TFpredict and SABINE: Sequence-Based Prediction of Structural and Functional Characteristics of Transcription Factors
Source: PLoS One. 2013 Dec 12;8(12):e82238. doi: 10.1371/journal.pone.0082238 (PMC3861411; doi:10.1371/journal.pone.0082238)

Figure S3: Regression error of SABINE SVR models

Shown is the **(A)** mean squared error (MSE) and the **(B)** average absolute error (AAE) of the support vector regression models used by SABINE to predict the PFM similarity of two TFs based on diverse features measuring the sequence similarity of their DNA-binding domains. SVR models were trained separately for each of the 5 structural superclasses, based on TF pairs with a DNA-binding domain sequence similarity > 0.3 with respect to the BLOSUM62 substitution matrix. Then, the regression error, i.e., the difference between the true and the predicted PFM similarity of each TF pair was assessed by cross-validation.

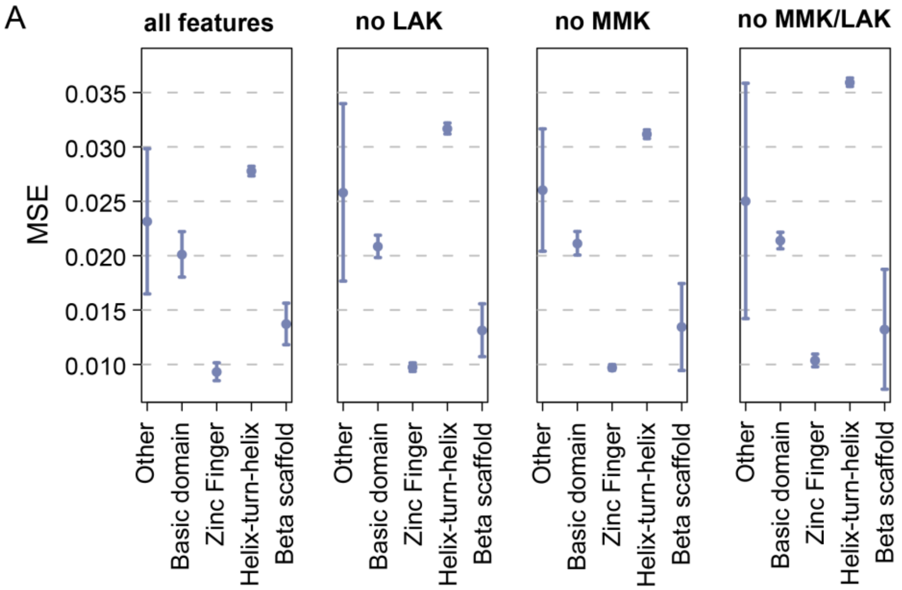

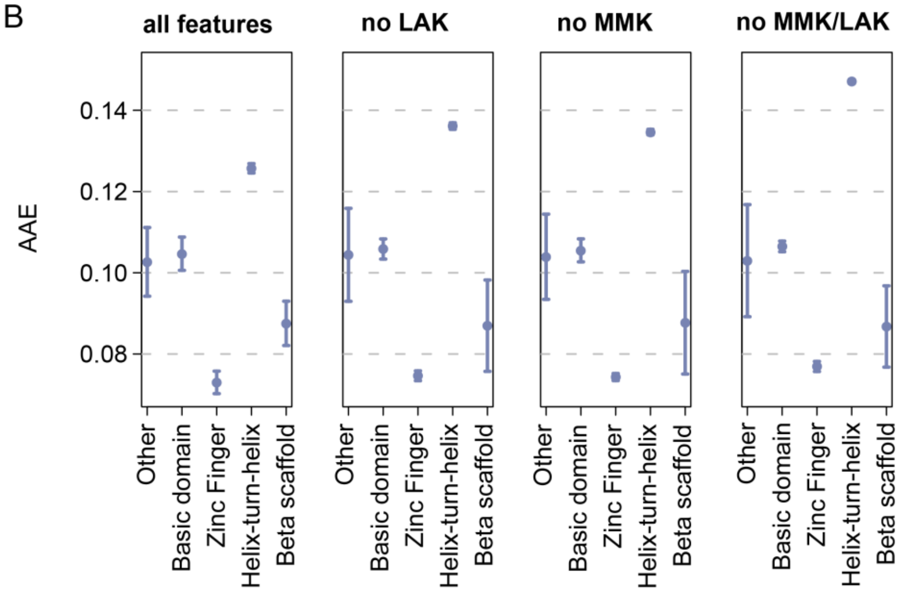

Supplement: Figure S3 — Regression error of SABINE SVR models. Shown is the (A) mean squared error (MSE) and the (B) average absolute error (AAE) of the support vector regression models used by SABINE to predict the PFM similarity of two TFs based on diverse features measuring the sequence similarity of their DNA-binding domains. SVR models were trained separately for each of the five structural superclasses, based on TF pairs with a DNA-binding domain sequence similarity >0.3 with respect to the BLOSUM62 substitution matrix. Then, the regression error, i.e., the difference between the true and the predicted PFM similarity of each TF pair was assessed by cross-validation. (PDF) [file pone.0082238.s003.pdf]
